# Enhancement of targeted therapy in combination with metformin on human breast cancer cell lines

**DOI:** 10.1186/s12964-023-01446-0

**Published:** 2024-01-02

**Authors:** Ghazal Mahmoudi, Yahya Ehteshaminia, Parviz Kokhaei, Seyedeh Farzaneh Jalali, Farhad Jadidi-Niaragh, Abdol Sattar Pagheh, Seyed Ehsan Enderami, Saeid Abedian Kenari, Hadi Hassannia

**Affiliations:** 1 Student Research Committee, Amol School of Paramedical Sciences, Mazandaran University of Medical Sciences, Sari, Iran; 2 Department of Immunology, School of Public Health, Tehran University of Medical Sciences, Tehran, Iran; 3 Department of Immunology, Arak University of Medical Sciences, Arak, Iran; 4 Department of Hematology, Faculty of Allied Medicine, Kerman University of Medical Sciences, Kerman, Iran; 5 Immunology Research Center, Tabriz University of Medical Sciences, Tabriz, Iran; 6 Infectious Diseases Research Center, Birjand University of Medical Science, Birjand, Iran; 7 Immunogenetics Research Center, School of Medicine, Mazandaran University of Medical Sciences, Sari, Iran; 8 Department of Paramedicine, Amol School of Paramedical Sciences, Mazandaran University of Medical Sciences, Sari, Iran

**Keywords:** Breast cancer, Combination therapy, Targeted therapy, Metformin

## Abstract

**Background:**

Breast cancer remains a primary global health concern due to its limited treatment options, frequent disease recurrence, and high rates of morbidity and mortality. Thereby, there is a need for more effective treatment approaches. The proposal suggests that the combination of targeted therapy with other antitumoral agents could potentially address drug resistance. In this study, we examined the antitumoral effect of combining metformin, an antidiabetic drug, with targeted therapies, including tamoxifen for estrogen receptor-positive (MCF-7), trastuzumab for HER2-positive (SKBR-3), and antibody against ROR1 receptor for triple-negative breast cancer (MDA-MB-231).

**Methods:**

Once the expression of relevant receptors on each cell line was confirmed and appropriate drug concentrations were selected through cytotoxicity assays, the antitumor effects of both monotherapy and combination therapy on colony formation, migration, invasion were assessed in in vitro as well as tumor area and metastatic potential in ex ovo Chick chorioallantoic membrane (CAM) models.

**Results:**

The results exhibited the enhanced effects of tamoxifen when combined with targeted therapy. This combination effectively inhibited cell growth, colony formation, migration, and invasion in vitro. Additionally, it significantly reduced tumor size and metastatic potential in an ex ovo CAM model.

**Conclusions:**

The findings indicate that a favorable strategy to enhance the efficacy of breast cancer treatment would be to combine metformin with targeted therapies.

**Graphical Abstract:**

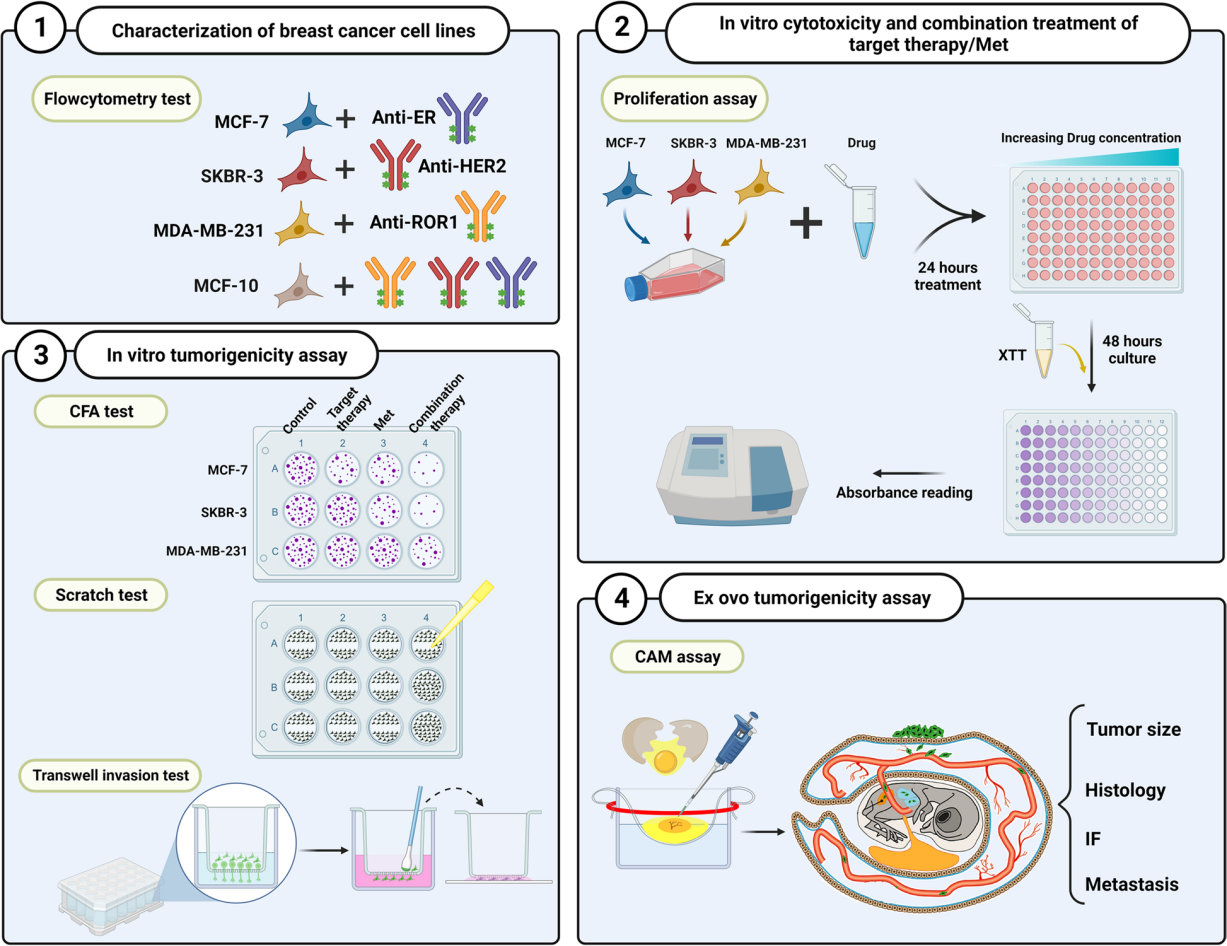

## Introduction

Despite extensive efforts, breast cancer continues to be a significant cause of morbidity and mortality on a global scale. In 2020, there were an estimated 2.3 million new cases of breast cancer and 680,000 deaths attributed to the disease [1]. The recurrence of tumor cells in vital organs is a critical issue and the second leading cause of death in women with breast cancer [2]. Surgery, chemotherapy, and radiotherapy are commonly used to treat breast cancer. However, disease replacements resulting from relict tumor cells and metastasis are frequently associated with substantial treatment side effects [3, 4]. In recent years, the fourth type of therapy, i.e., targeted therapy (hormone therapy and antibody therapy), has proved more effective in treating breast cancer subtypes [5]. Breast cancer is mainly classified into three subtypes based on the presence or absence of estrogen receptor (ER), progesterone receptor (PR), and human epidermal growth factor receptor 2 (HER2). They include high expression of estrogen and progesterone receptor (ER + , PR +), HER2 + (HER2 high/overexpressed), and triple-negative breast cancers (TNBC; ER-, PR-, HER2-) [6]. The benefit of this particular treatment is its ability to specifically target cancer cells, resulting in minimal side effects [7].

Currently, the exclusive treatments for ER + and HER2 + breast cancer subtypes are tamoxifen (Tam) [6] and trastuzumab (Tras), respectively. There is currently no effective targeted treatment available for triple-negative breast cancer. As a result, first-line therapies for TNBC patients are currently limited to traditional chemotherapy by paclitaxel and cisplatin drugs that are highly cytotoxic and long-term therapy using these treatments may lead to drug resistance and disease recurrence [8]. Therefore, it is necessary to identify effective treatments that can overcome drug resistance [5]. Recent studies have shown that the Receptor tyrosine kinase-like orphan receptor 1 (ROR1) is highly expressed in TNBC, making it a potential candidate for targeted therapy in this type of breast cancer [9]. Cancer combination therapy is considered to be an appropriate approach for overcoming resistance. This therapeutic method offers several benefits compared to traditional treatments, such as reduced toxicity, improved effectiveness, and lower dosage requirements, while maintaining an equal or greater level of impact in oncological therapy [10, 11].

Thanks to prior knowledge of their pharmacokinetics, dynamics, and toxicities, there is a strong interest in utilizing non-cancer drugs for cancer treatment [12]. Numerous studies have demonstrated that Metformin (Met), a widely used type 2 antidiabetic medication, possesses antitumoral properties. It has been shown to effectively inhibit the growth of cancer cells and decrease the likelihood of developing solid tumors, including ovarian, colon, and breast cancer [13–15]. Furthermore, studies focusing on combination therapy have demonstrated the reinforcing effects of Met combination therapy in pancreatic, lung, and breast cancers [16–18]. In this study, we aim to investigate the effects of combining Met therapy with targeted therapy on different types of breast cancer cell lines.

## Material and methods

### Materials

Dulbecco’s modified medium (DMEM) (Sigma-Aldrich, USA), Heat-inactivated fetal bovine serum (FBS) (Gibco, USA), Penicillin (Gibco, USA), Streptomycin (Gibco, USA), Metformin (Sigma-Aldrich, USA), Tamoxifen (Sigma-Aldrich, USA), Trastuzumab (Roche, Switzerland), Anti-ROR1 antibody (Clone 2A2, Biolegend, USA).

### Cell lines and cell culture

The human breast cancer cell lines MCF-7, SKBR-3, and MDA-MB-231 and the non-cancerous epithelial MCF-10A breast cell line were obtained from the Pastor Institute's National Cell Bank in Tehran, Iran. The cells were cultured in DMEM medium, which was supplemented with 10% heat-inactivated fetal bovine serum (FBS) and 1% penicillin/streptomycin (100 units/mL). The cells were kept at 37°C in a 5% CO_2_ humidified atmosphere. A PCR Mycoplasma Detection Kit (in-house) was used to confirm that all cultured cells are negative for mycoplasma contamination. The experimental procedures were conducted per the guidelines provided by the Ethical Committee of Mazandaran University of Medical Sciences (IR.MAZUMS.REC.1398.6923).

### Flow cytometry

Flow cytometry was employed to assess the expression of ER, HER2, and ROR1 proteins on each tumor cell line. This involved direct staining of fluorochrome-conjugated antibodies and isotype-matched fluorochrome-labeled control mAbs (BioLegend or BD Biosciences, San Jose, CA, San Diego, CA), as previously described [19]. In order to perform intracellular ER staining, the cells were initially permeabilized with a BD Cytofix/Cytoperm solution for 30 min at 4°C. Flow cytometry was performed using a FACS Calibur instrument (Becton-Dickenson, Mountain View, CA, USA). The data were subsequently analyzed with FlowJo_V7 software (Tree Star Inc., Ashland, OR, USA).

### In vitro cytotoxicity assay

The XTT assay was used to assess cell viability following targeted therapy and Met, either alone or in combination, in both human cancerous and non-cancerous breast cell lines. Briefly, for targeted therapy, the MCF-7 cells (8 × 10^3^ cells/well) were treated with various concentrations of Tam (0.1–10 µM). Similarly, the SKBR-3 cells (6 × 10^3^ cells/well) were treated with different concentrations of Tras (0.01–50 µg/ml), and the MDA-MB-231 cells (8 × 10^3^ cells/well) were treated with varying concentrations of anti-ROR1 (0.1–10 µg/ml). For combination therapy, each cell line was treated with Met at different concentrations (2.5–20 mM). Additionally, MCF-10A cells, serving as the normal control, were subjected to specific doses of Met (5 mM), Tam (1 µM), Tras (0.1 µg/ml), and anti-ROR1 (10 µg/ml). After the cells were incubated for 48 h at 37 °C, the XTT solution (Sigma-Aldrich, USA) was added to all wells and incubated for 2 h. The optical density was measured at 450 nm using an ELISA reader (Biotech Instruments, INC, USA), with a reference wavelength of 650 nm [19]. The percentage of cell viability and inhibitory concentrations (IC_20_ and IC_50_) were determined by constructing a dose–response curve using GraphPad Prism software version 9 (San Jose, CA, USA).

### Colony formation assay (CFA)

The breast cancer cell lines (3 × 10^3^ cells/well) were plated in a 6-well plate. After overnight incubation, the cells were treated with a select minimum dose of Tam (1µM), Tras (0.1 µg/ml), and anti-ROR1 (10 µg/ml), either alone or in combination with Met (5mM). The plates were incubated for 7 days to allow colony formation. The colonies were fixed with methanol and subsequently stained with crystal violet. Finally, the colonies with more than 10 cells were manually counted under an inverted light microscope (magnification: × 40; Labomed Inc., USA). The rate of clone formation was then calculated using the following formula:

$$\mathrm{Plate}\;\mathrm{clone}\;\mathrm{formation}\;\mathrm{rate}\;(\%)\:=\:(\mathrm{number}\;\mathrm{of}\;\mathrm{treated}\;\mathrm{well}\;\mathrm{clones}/\mathrm{number}\;\mathrm{of}\;\mathrm{control}\;\mathrm{well}\;\mathrm{clones})\:\times\:100\%$$

where A is the width of the cellular motility before incubation, and B is the width of the cell motility after incubation.

### Migration assay

In order to perform the scratch assay, the cells were cultured until they reached confluence (~ 90%). A 100-μl pipette tip was used to create a scratch. After the scratch was made, each well was washed with PBS to remove any unattached cells or debris. The cells were maintained in predefined media (1 µM Tam, 0.1 µg/mL Tras, and 10 µg/mL anti ROR) alone or combined with 5 mM Met for 48 h. The images were captured at 0 h and 48 h using an inverted light microscope to assess the cell-migrating ability. The area within the gap was calculated using Image J software (version 1.53 NIH, Bethesda, MD, USA) based on the following formula:

$$\mathrm{Migration}\;\mathrm{area}\;(\%)\:=\:100\;(\mathrm{AX}\:-\:\mathrm{BX})/(\mathrm A\;\mathrm{blank}\:-\:\mathrm B\;\mathrm{blank}),$$

### Transwell invasion assay

The breast cancer cells were deprived for 24 h in serum-free DMEM medium prior to the experiment. The cells were subsequently placed in the upper chambers of 24-well invasion plates. These plates featured a Matrigel-coated membrane with 8 μm pores (Millipore, MA, USA) and a density of 25,000 cells per well. The lower chambers contained DMEM supplemented with 5% FBS, serving as a chemoattractant. The cells were treated with the predefined concentration of targeted therapy and/or Met, as described above. After a 48-h incubation period, the breast cancer cells that remained on the upper side of the invasion filter were meticulously removed by gently scraping them off using a pre-wet cotton swab. The invading cells, which had passed through the filter and reached the lower surface, were fixed in methanol and then stained with 1% crystal violet. The filters were subsequently removed and carefully mounted onto glass slides. The invading cells were counted in five random fields using a BX41 microscope (Olympus, Tokyo, Japan) and expressed as a percentage of the corresponding controls.

### Chick chorioallantoic membrane (CAM) assay

To evaluate the potential of combination therapy in inhibiting tumorigenicity, we performed the ex ovo CAM assay as described by Deryugina et al. (15): The fertilized chicken eggs were obtained from a local provider in Amol, Mazandaran, Iran. The eggs were incubated horizontally, with intermittent rotation, at a temperature of 37.5°C and a humidity level of 65% for 3 days. Once the eggshells cracked, the embryos were transferred into small, sterile plastic bowls. The bowls were covered with Petri dishes and incubated. On day 7 of embryonic development, the silicon rings were placed on the CAM, and 1 × 10^6^ tumor cells were seeded on the rings. After nine days, when the tumor developed, the selected doses of monotherapy and combination therapy were administered on rings to restrict treatment zones. On day 14, embryos were sacrificed, and xenograft tumors were retrieved and photographed using a stereomicroscope (Labomed CZM6, Labo America Inc., USA) to measure their area. The tumor area was determined by inserstion of the sagittal and transversal diameters in the following ellipsis formula: (A = Pi × d1[sagittal] × d2[transversal]). For further histological analysis, the tumors were fixed with formaldehyde and subjected to immunofluorescence [19].

### Analysis of tumor cells metastasis in CAMs by quantitative PCR (qPCR)

After the ex ovo CAM assay was terminated on day 14, the lungs and livers of the chicks were harvested and analyzed for human genomic DNA using quantitative Alu PCR in order to determine the incidence of tumor metastases in each group. In brief, total DNA was extracted from the tissues using the DNA extraction kit (Favorgen, Taiwan). Moreover, chicken glyceraldehyde 3-phosphate dehydrogenase (GAPDH) was used as an endogenous control to ensure that the DNA input for the qPCR reactions was standardized. A standard curve was subsequently generated by performing serial dilutions of breast cancer cell line DNA, ranging from 5 × 10^2^ to 5 × 10^6^ cells/mL. The quantity of the extracted DNA was determined by spectrophotometry at a wavelength of 260 nm in order to assess both its purity and concentration. Alu sequences were quantified using specific primers via qPCR [20]. The threshold cycle values were plotted on the standard curve to ascertain the quantity of human tumor cells in each treatment group.

### Statistical analysis

The statistical analyses in this study were performed using the SPSS statistical package (SPSS, Chicago, IL, USA) and GraphPad Prism (San Diego, California, USA). Two group comparisons were assessed using a Mann–Whitney U test, while comparisons between groups were investigated through one-way ANOVA analysis. All experiments were performed in triplicate. The data is presented as the mean ± standard deviation (SD). Statistical significance was considered at * *P* < 0.05, ** *P* < 0.01 and *** *P* < 0.001.

## Results

### Expression of ER, HER2, and ROR1 on breast cancer cell lines

**Fig. 1:**
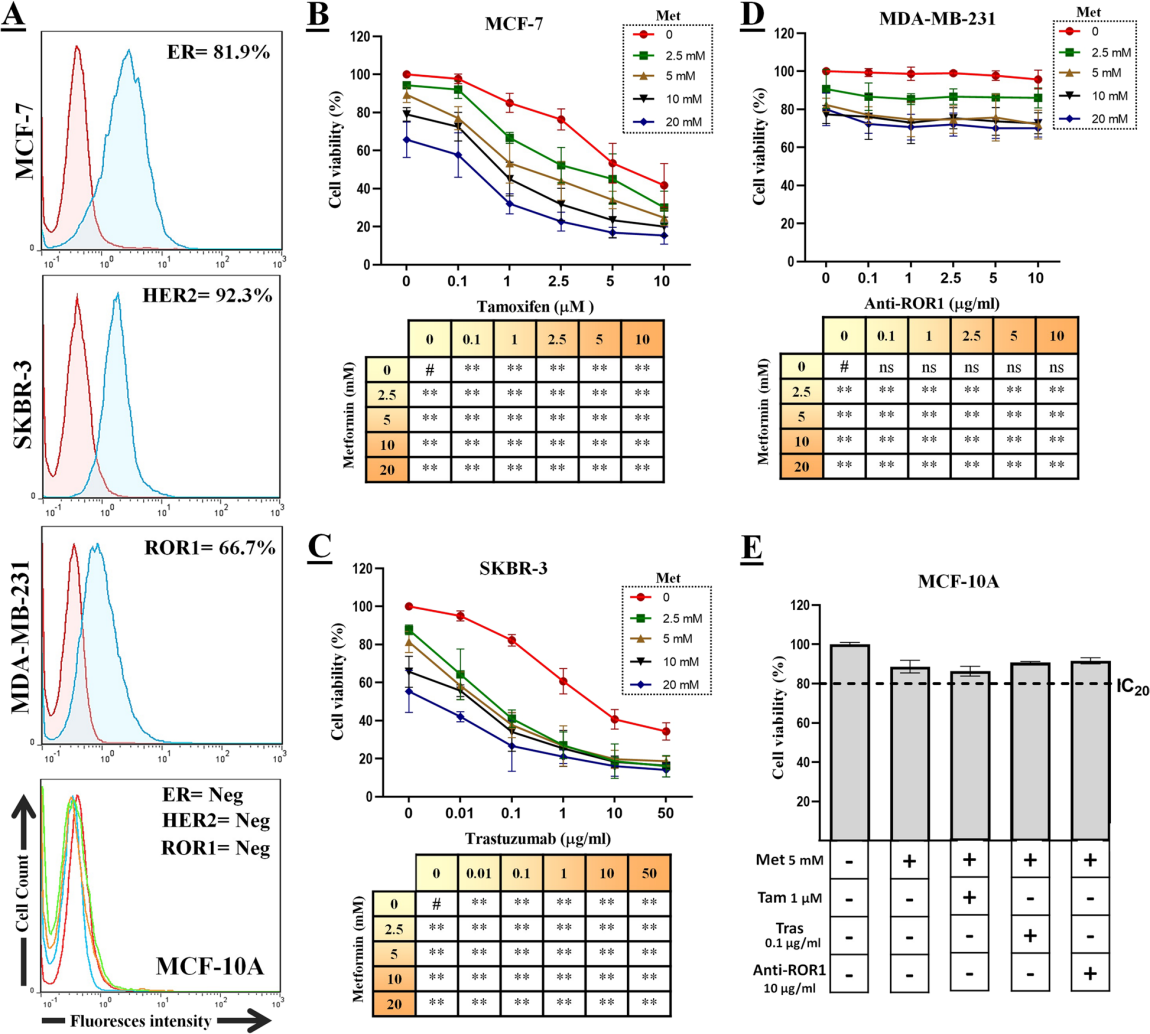
Analysis of ER, HER2, and ROR1 expressions and cytotoxicity assay in various types of breast cancer and the normal cell line. **A** Flow cytometry was examined to confirm the expression of receptors on each breast cancer cell line. Histograms represent the fluorescence signals (blue) with an isotype control (red). **B**-**D** MCF-7, SKBR-3, and MDA-MB-231 cells were treated with different concentrations of either Tam (0.1–10 µM), Tras (0.01-50µg/ml), and anti-ROR1 (0.1–10 µg/ml) or Met at different concentrations (2.5–20 mM), respectively. Then, the cytotoxicity curve was drawn for each cell line. **E** MCF-10A cells as a normal control were treated with selected doses of Met (5mM), Tam (1µM), Tras (0.1µg/ml), and anti-ROR1 (10 µg/ml). The *p* values were determined by Mann–Whitney U test for each treatment group and compared to the control group. The data analysis was represented as the mean ± SD. ns = not significant and **P < 0.01

Flow cytometry was utilized to validate the expression of receptors for targeted therapy. The expression level of ER on MCF-7 was 81.9%, HER-2 on SKBR3 was 92.3%, and ROR1 on MDA-MB-231 was 66.7%, as depicted in Fig. 1A. In addition, ER, HER2, and ROR1 expressions in MCF-10A cells (normal breast epithelium) as a control group were all negative.

### Cytotoxic effects of targeted therapy and met

In order to assess the impact of combination treatment on the viability of breast cancer cells, a cytotoxicity curve was generated using the XTT assay. The MCF-7 cells were treated with various concentrations of either Tam, or Met. The results demonstrated that different concentrations of Tam and Met inhibit MCF-7 cell growth in a dose-dependent manner (Fig. 1B). Similar results were obtained for the inhibitory effects of Tras and Met on SKBR-3 cells (Fig. 1C). However, it is evident that anti-ROR1 had no significant impact on the viability of the MDA-MB-231 lineage, regardless of the varying concentrations used (Fig. 1D).

Moreover, IC_20_ values for Tam and Met were measured based on a dose–response curve at concentrations of 2.2 µM and 9.3 mM, respectively. Additionally, Tam was able to inhibit the growth of MCF-7 cells by 50% (IC_50_) at a concentration of 6.4 µM. Additionally, Tras and Met inhibited SKBR-3 cell growth by 20% (IC_20_) at a concentration of 0.14 µg/ml and 5.4 mM, respectively. The IC_50_ value for Tras was measured at a concentration of 1.1 µg/ml in SKBR-3 cells. On the other hand, Met demonstrated the ability to hinder cell growth of MDA-MB-231 cells, with an IC_20_ value of 7.1 mM. It should be noted that the IC_20_ and IC_50_ values related to MDA-MB-231 and the IC_50_ value for Metformin in all cell lines have not been determined.

In order to investigate the alterations in tumor cell behavior and their corresponding molecular signaling, we opted for a concentration below IC_20_ (where 80% of cells remain viable and the observed effect is not attributed to cell death). After evaluating IC_20_ for targeted therapy and Met, we confirmed the non-toxic effects of the selected doses by utilizing MCF-10A cells as a normal cell lineage (Fig. 1E).

### Met combination therapy strongly inhibits tumor colonization, migration, and invasion

**Fig. 2:**
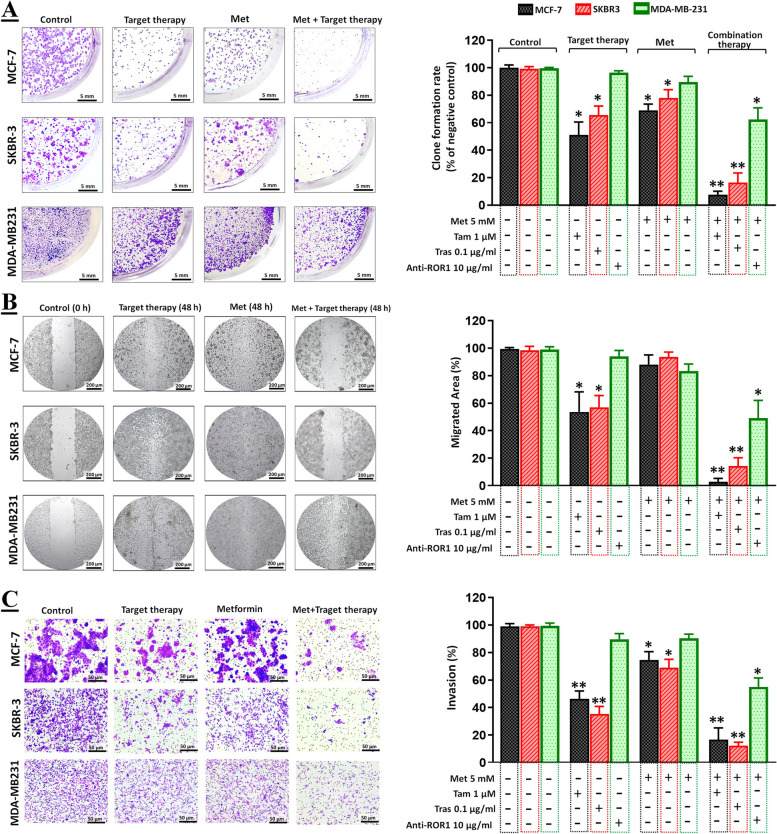
The impact of the combination of targeted therapy and Met on the CFA, migration, and invasion of breast cancer cell lines. **A** The CFA test was conducted in order to evaluate the formation of colonies before and after treatment. The cells were treated with a selected dose of Tam (1µM), Tras (0.1 µg/ml), and anti-ROR1 (10 µg/ml), either alone or in combination with Met (5mM). **B** The migration assay was conducted to assess the influence of different treatments on cell migration. The treatment groups received selected doses as described above and the cell density of clones observed under inverted microscope (magnification: × 40, scale bar: 200 µm). **C** The invasion assay was conducted to evaluate the impact of different treatments on cell invasion. The treatment groups received selected doses and the images were captured (magnification: × 40, scale bar: 50 µm). Graphs represent the result analysis of each test (on the right). Data is represented as the mean ± SD. *P < 0.05 and **P < 0.01

In order to evaluate the tumorigenicity of breast cancer cell lines in vitro, the first step involved performing a CFA to assess the reproductive capacity of the cells following treatment. As depicted in Fig. 2A, the number of MCF-7 and SKBR-3 colonies decreased significantly following monotherapy (targeted therapy/Met) and combination therapy. However, the MDA-MB-231 cells did not exhibit a remarkable response to monotherapy (targeted therapy/Met). Only the combination therapy effectively reduced the number of colonies compared to the control group. Indeed, combination therapy was approximately seven-, four-, and 1.5-fold more effective than targeted therapy alone at reducing cell reproductive capacity in MCF-7, SKBR-3, and MDA-MB-231 cells, respectively.

Furthermore, migration and invasion assays were conducted to assess the efficacy of various therapies in inhibiting metastatic processes. As observed in Fig. 2B and C, the migration and invasion capacity of MCF-7 and SKBR-3 cells were significantly reduced after monotherapy (targeted therapy/Met) and combination therapy. However, the MDA-MB-231 cells did not respond significantly to monotherapy (targeted therapy/Met). Only the combination therapy was able to effectively reduce cell migration and invasion when compared to the control group. Therefore, the combination therapy was approximately nineteen times, four times, and two times more effective than targeted therapy alone at reducing cell migration in MCF-7, SKBR-3, and MDA-MB-231 cells, respectively. Additionally, the combination therapy resulted in approximately a three-fold greater inhibition of cell invasion on MCF-7 and SKBR-3 cells and a two-fold greater inhibition on MDA-MB-231 cells compared to targeted therapy alone.

### Antitumor effects of monotherapy and combination therapy in ex ovo CAM assay

**Fig. 3:**
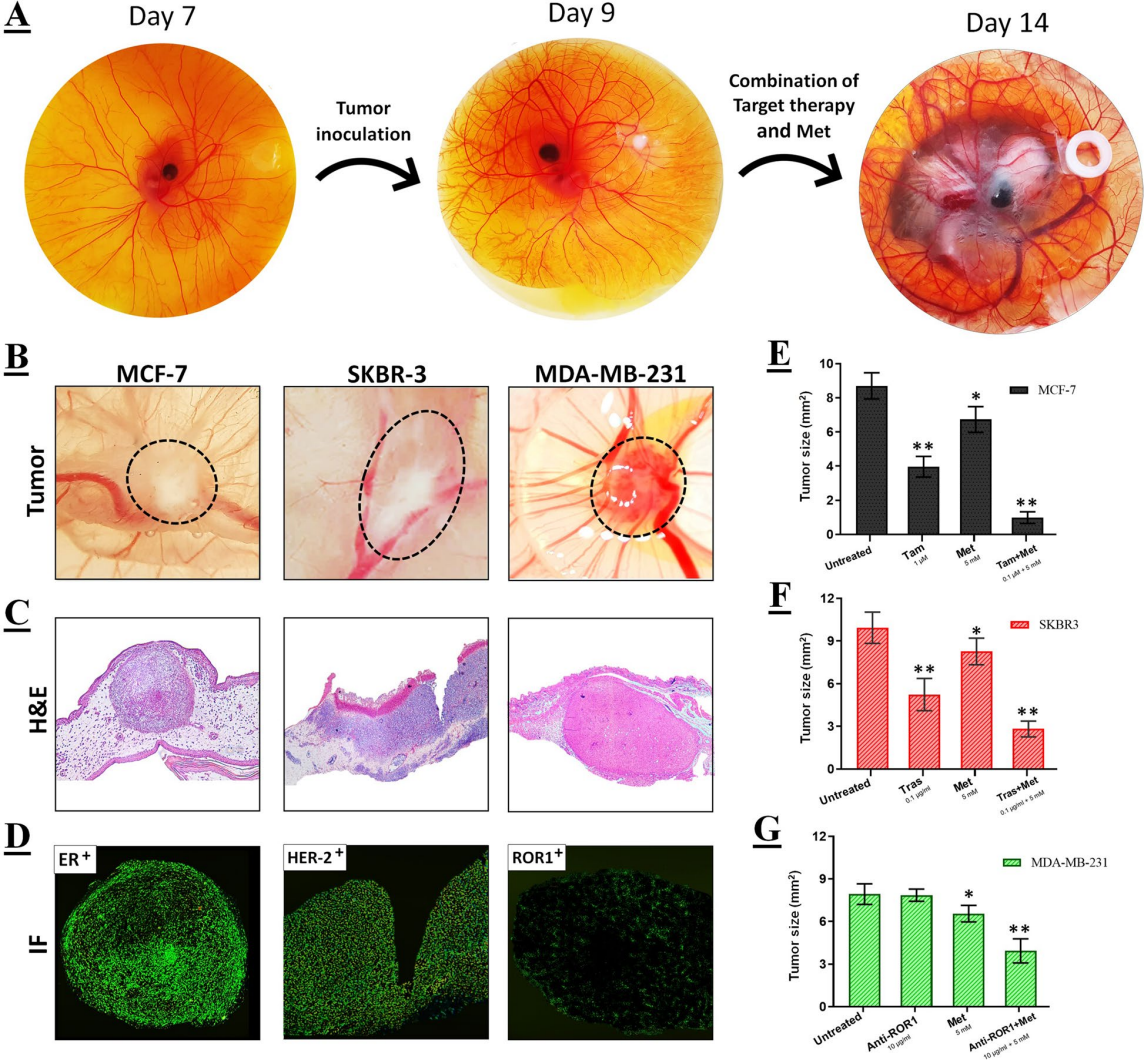
The chorioallantoic membrane (CAM) assay. **A** In order to outline the procedure, the tumor cells were placed onto the CAM after a 7-day period. When the tumor developed, it was treated using the predefined concentration targeted therapy or targeted therapy with Met. After 7 days, all xenograft tumors were gently incised and observed to have enlarged. **B** Tumor area was shown in untreated groups. **C** Photo documentation of tumor development histology was demonstrated in untreated groups. **D** IF assay was conducted to confirm the target proteins’ expression on CAM assay in untreated control groups. **E**–**G** the graphs depict the quantification of tumor area (mm2) in untreated, monotherapy, and combination therapy cells for MCF-7 (ER +), SKBR-3 (HER2 +), and MDA-MB-231 (ROR1 +) respectively. Data is represented as the mean ± SD. *P < 0.05 and **P < 0.01

To validate the in vitro results, we assessed the antitumor effects of both monotherapy and combination therapy in the xenogeneic ex ovo model. The diagram illustrating the ex ovo assay technique is depicted in Fig. 3A. As previously mentioned in the methods section, the tumors were excised on day 14 and subsequently photographed (Fig. 3B). To validate the findings of the CAM assay, the tumors were subsequently fixed for histological analysis (Fig. 3C). The control group of MCF-7, SKBR-3, and MDA-MB-231 tumors exhibited an increased rate of cell division and proliferation (an average of 3–5 mitosis/high-power field [hpf]). In the monotherapy group, however, a low rate of mitosis (2–4 mitosis/hpf) was observed. Surprisingly, the mitotic rate in the combination therapy group was extremely low in all three cell lines (> 1 mitosis/5 hpf). All treatments resulted in a chaotic and less compact tissue organization with dispersed and difficult-to-identify cells, despite the fact that tumors treated with the combination therapy exhibited no apparent damage to tissue structure and had a homogeneous cell population in morphology and distribution.

Additionally, to verify the target proteins' expression on CAM assay, we performed an IF assay (Fig. 3D). The results showed that, MCF-7 and SKBR-3 cells had high levels of ER and HER2 receptors. However, the expression of the ROR1 receptor was low and varied among different MDA-MB-231 cells. Furthermore, as depicted in Fig. 3E, the combination of Tam and Met effectively suppressed the growth of MCF-7 tumors. The tumor area was reduced by approximately 89% when compared to the untreated control cells. Similar results were obtained for SKBR-3 tumors when the combination of Tras and Met was utilized (Fig. 3F). The area of these tumors was reduced by approximately 72% compared to untreated cells. According to Fig. 3G, the growth of MDA-MB-231 tumors was inhibited by the combination of anti-ROR1 and Met. The tumor area was reduced by approximately 51% compared to the untreated and targeted therapy alone.

### Anti-metastatic effects of monotherapy and combination therapy in ex ovo CAM model

**Fig. 4:**
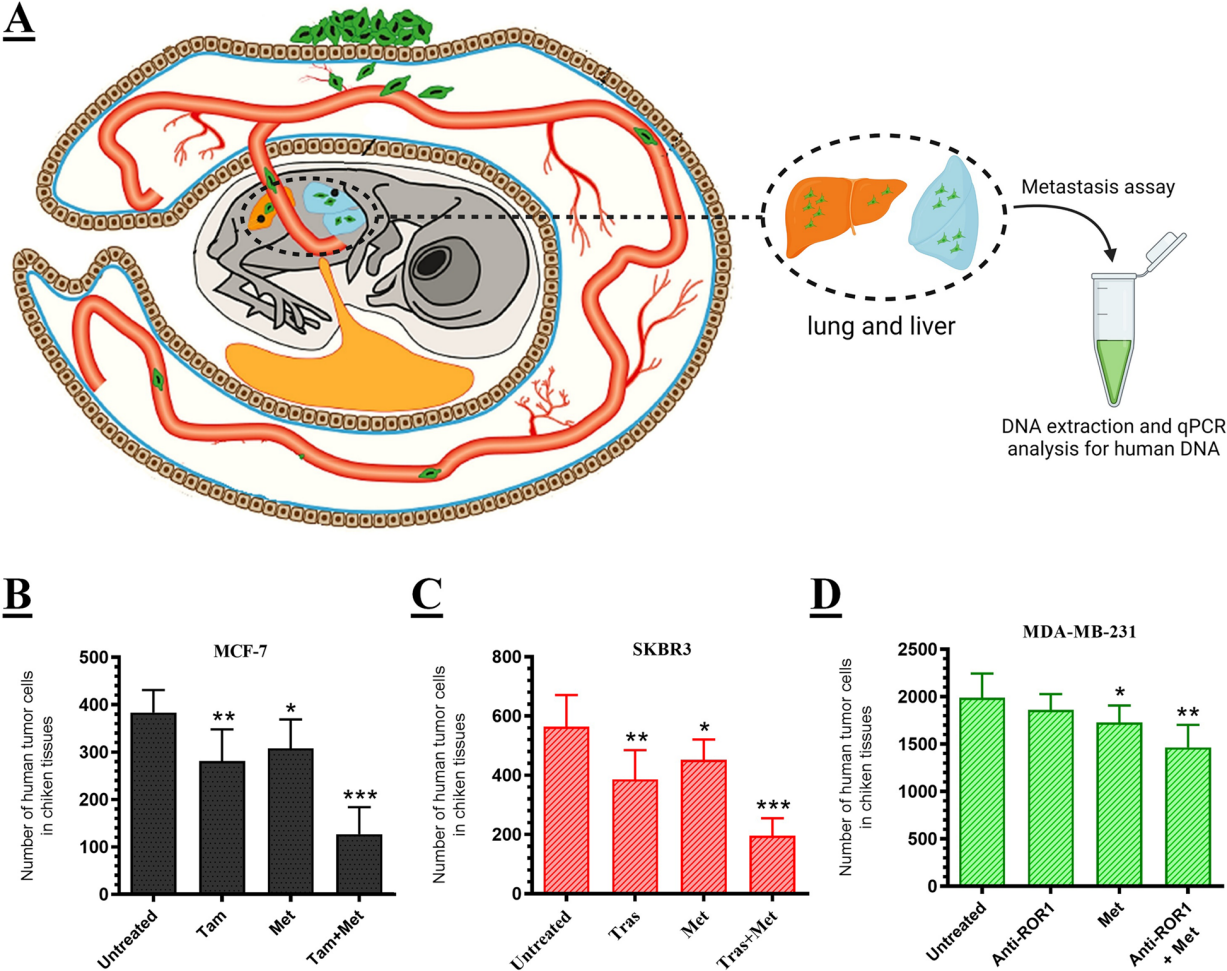
The influence of the combination of targeted therapy and Met on the breast cancer tumors metastasis. **A** The schematic diagram of metastasis is illustrated in the ex ovo CAM model. Graphs represent the quantitative PCR analysis of MCF-7 **B**, SKBR-3 **C**, and MDA-MB-231 **D** xenograft tumors. The quantitative PCR method was utilized to specifically target Alu sequences of human DNA in chick tissues. The presence of equivalent quantities of host genomic DNA was confirmed using quantitative real-time PCR of chGAPDH. Data is represented as the mean ± SD. *P < 0.05 and **P < 0.01

In order to evaluate the impact of monotherapy and combination therapy on tumor metastasis, we assessed the human genome in chick tissues (Fig. 4A) [21]. As depicted in Fig. 4B, the concentration of human DNA was significantly lower in MCF-7 across all treated groups compared to the control group. However, when Tam and Met were combined, there was a significant decrease of approximately 67% in the human DNA concentration within embryos compared to the untreated control group. A similar reduction was observed in the groups treated with SKBR-3 (Fig. 4C). However, the combination of Tras and Met resulted in a significant decrease in human DNA concentration, reducing it by approximately 65% compared to the untreated control group. Furthermore, the levels of human DNA in embryos treated with anti-ROR1 and Met were reduced by approximately 27% compared to embryos that were untreated or received monotherapy alone (Fig. 4D).

## Discussion

Recent advancements in cancer treatment are shifting towards non-toxic, specific, and low side-effect methods, specifically targeted therapy. Despite the interest in targeted therapy, the main challenges of this treatment remain resistance, metastasis, and different responses in patients [22, 23]. Combination therapy has been demonstrated as an appropriate approach to cancer treatment [24]. The antitumor activity of Met, which is an antidiabetic drug that affects various metabolic parameters, has recently been demonstrated [25, 26]. In this study, we assessed the impact of Met in conjunction with targeted therapy on various breast cancer cell lines. First, we confirmed the presence of ER, HER2, and ROR1 proteins on each tumor cell line through flow cytometry analysis (Fig. 1A). Next, a cytotoxicity curve was plotted to determine the appropriate doses of Met and targeted therapy. The results indicate that Met augmented the inhibitory effects of Tam (Fig. 1B), Tras (Fig. 1C), and anti-ROR1 (Fig. 1D) on MCF-7, SKBR-3, and MDA-MB-231 cells, respectively.

To assess the alterations in functional behavior and associated signaling pathways of tumor cells, we opted for a concentration below IC_20_ for the subsequent experiments. Based on the obtained results, we chose a concentration of 5mM Met in combination with 1µM Tam, 0.1µg/ml Tras, and 10µg/ml anti-ROR1. Additionally, the non-toxic impact of the selected doses was confirmed using MCF-10A cells (Fig. 1E). Our findings were consistent with previous studies that have shown the inhibitory effect of Met at a concentration of 50 mM on the viability of MCF-7, MCF-7/713, BT-474, and SKBR-3 cells [27]. We conducted further investigations to determine the potential impact of a combination of targeted therapy and Met on the CFA, migration, and invasion of breast cancer cells. The results of the CFA indicate that when targeted therapy and Met are present at non-toxic concentrations, they significantly inhibit the reproductive capacity of individual breast cancer cell lines (Fig. 2A). However, the combination of targeted therapy and Met has a significant contribution to reducing the colony formation of breast cancer cell lines. Besides, the results of the migration and invasion experiments revealed that the combination of targeted therapy and Met effectively decreased the migratory and invasive abilities of breast cancer cells, as compared to monotherapy (Fig. 2B and C).

Previous studies have demonstrated the inhibitory effect of Met on colony formation, migration, and invasion, which aligns with our findings. This is supported by a study conducted by Kim et al., which revealed the inhibitory influence of Met on colony formation in breast cancer cells [28]. Another study illustrated that treatment with Met effectively decreased the migration and invasion of MDA-MB-231 cells [29].

Zhang J et al. demonstrated the inhibitory effect of Met on ER-positive and triple-negative breast cancer cell lines migration and invasion [30]. The study conducted by Bing Cui et al. found that anti-ROR1 exhibited a significant response in inhibiting the migration and metastatic capacity of MDA-MB-231 cells [31]. This contradiction may have several probable reasons. First, some of their research was conducted in an in vivo environment, where mechanisms such as complement-dependent cytotoxicity (CDC) and antibody-dependent cellular cytotoxicity (ADCC) may enhance antibody function. Additionally, as depicted in Fig. 1A, the ROR1 protein expression on MDA-MB-231 cells was approximately 66.7%; and nearly 33% of the cells displayed no detectable ROR1 expression, making the antibodies ineffective in their targeting. Furthermore, among the ROR1-positive cell population, ROR1 demonstrated diverse surface expressions, with notably low mean fluorescence intensity (Fig. 3D).

Also, the impact of the combination of targeted therapy and Met on the tumorigenicity of human breast xenograft tumors in the chicken embryo model was assessed using the ex ovo CAM assay (Fig. 3A and B). The CAM results were confirmed through histological analysis, and the expression of target proteins was assessed using IF analysis (Fig. 3C and D). The histological outcomes indicate that non-toxic monotherapy concentrations decreased the mitosis rate in MCF-7, SKBR-3, and MDA-MB-231 cells. However, combination therapy significantly reduced the miotic rate of all three cell lines. Furthermore, the results regarding tumor area indicate that the combination of targeted therapy and Met led to a significant reduction in the area of MCF-7 (Fig. 3E), SKBR-3 (Fig. 3F), and MDA-MB-231 (Fig. 3G) tumors compared to untreated control cells or monotherapy alone. These findings suggest that combination therapy has enhanced effects in inhibiting tumor growth.

**Fig. 5:**
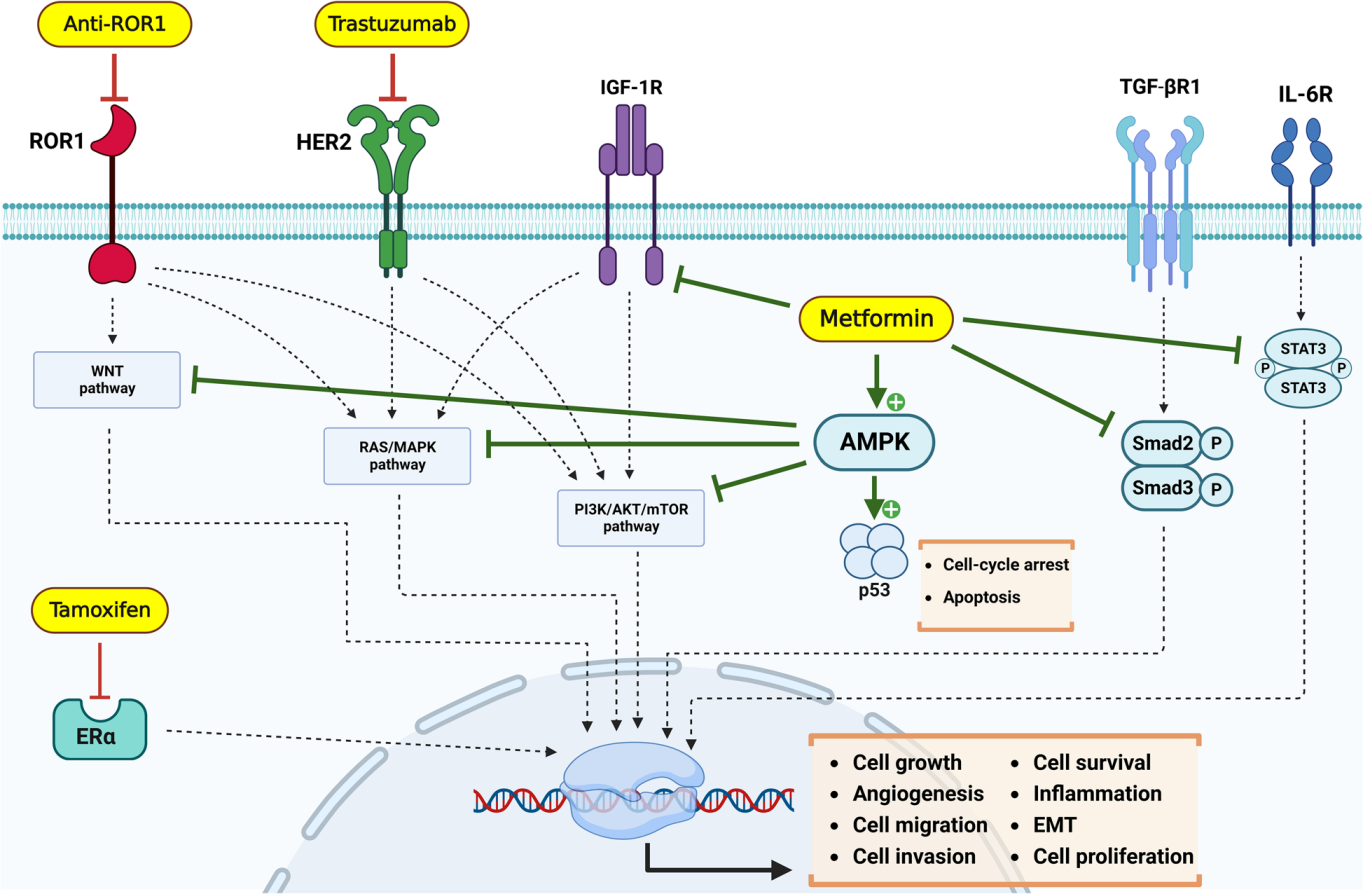
Proposed mechanism of action of Met to enhance targeted therapy in breast cancer treatment. Met activates AMPK, resulting in the inhibition of the PI3K/AKT/mTOR, RAS/MAPK, and WNT signaling pathways. It can also block P53 by activating AMPK, causing cell cycle arrest. In addition, Met can inhibit cytokine and growth factor receptors, including TGF-βR1, IL-6R, and IGF-1R, as well as their respective signaling pathways. In targeted therapy, Tam, Tras, and Anti-ROR1 are used to block the ER, HER2, and ROR1 receptors, respectively. Furthermore, the combination of targeted therapy and Met can enhance the inhibitory effect of existing drugs and have a synergistic impact on blocking the PI3K/AKT/mTOR, RAS/MAPK, and WNT pathways. All of these interactions lead to the inhibition of cell growth, angiogenesis, cell migration and invasion, inflammation, EMT, cell survival, and cell proliferation

We also assessed the impact of combining targeted therapy and Met on the metastasis of human breast tumors in the lungs and liver of chicken embryos (Fig. 4A). The results indicate that the combination therapy had a more significant effect on reducing human DNA concentrations in MCF-7 (Fig. 4B), SKBR-3 (Fig. 4C), and MDA-MB-231 (Fig. 4D) tumors compared to the other groups. Our findings are consistent with previous studies that have demonstrated the inhibitory effect of Met on metastatic capacity in mouse models. For instance, Wang et al. demonstrated the inhibitory effect of Met on the metastasis of 4T1 breast cancer cells [32]. The proposed mechanisms underlying the enhancing effect of Met on targeted therapy are illustrated in Fig. 5. One of the most important mechanisms is the activation of AMPK by Met, which leads to the blocking of the PI3K/AKT/mTOR, RAS/MAPK, and WNT signaling pathways. It can also block P53 by activating AMPK, resulting in cell cycle arrest. Additionally, Met has the ability to inhibit cytokine and growth factor receptors, including TGF-βR1, IL-6R, and IGF-1R, as well as their associated signaling pathways [33–35]. Further, in the context of targeted therapy, it is worth noting that Tam, Tras, and Anti-ROR1 can inhibit the ER, HER2, and ROR1 receptors, respectively [36–38]. Additionally, the combination of targeted therapy and Met can increase the inhibitory effect of existing drugs and produce an enhanced impact on the blockade of PI3K/AKT/mTOR, RAS/MAPK, and WNT pathways. These interactions result in the inhibition of cell growth, angiogenesis, cell migration, invasion, inflammation, EMT, cell survival, and cell proliferation.

## Conclusion

In this study, specific concentrations were selected for each drug, and the antitumor response was evaluated in in vitro and ex ovo models for three breast cancer subtypes. However, it is important to note that there are certain potential limitations to our study. First, we did not evaluate the direct influence of combination therapy on molecular signaling pathways, which is considered essential for future research. Secondly, non-toxic and lower concentrations of Met were utilized to assess the alterations in tumor cell behavior for the remaining experiments. However, higher concentrations may be employed in the mouse model. In sum, our findings demonstrate an enhanced anticancer effect of targeted therapy and Met in all subtypes of human breast cancer cells. These findings present a novel therapeutic approach that shows promise for potential future clinical trials.

## Data Availability

The participants of this study did not give written consent for their data to be shared publicly; therefore, supporting/raw data is not available.

## Abbreviations

ER: Estrogen receptor
PR: Progestrone receptor
HER2: Human epidermal growth factor receptor 2
ROR1: Receptor tyrosine kinase-like orphan receptor 1
TNBC: Triple negative breast cancer
Met: Metformin
FBS: Fetal bovine serum
IC_50_: 50% Inhibitory concentration
IC_20_: 20% Inhibitory concentration
CFA: Colony formation assay
CAM: Chick chorioallantoic membrane
qPCR: Quantitative polymerase chain reaction
hpf: High-power field
CDC: Complement-dependent cytotoxicity
ADCC: Antibody-dependent cellular cytotoxicity
